# Enhancing the Readability of Online Patient Education Materials Using Large Language Models: Cross-Sectional Study

**DOI:** 10.2196/69955

**Published:** 2025-06-04

**Authors:** John Will, Mahin Gupta, Jonah Zaretsky, Aliesha Dowlath, Paul Testa, Jonah Feldman

**Affiliations:** 1 Medical Center Information Technology Department of Health Informatics New York University Langone Health New York, NY United States; 2 Division of Hospital Medicine Department of Medicine New York University Langone Health New York, NY United States; 3 Ronald O. Perelman Department of Emergency Medicine New York University Grossman School of Medicine New York, NY United States; 4 Department of Medicine New York University Long Island School of Medicine Mineola, NY United States

**Keywords:** patient education, health literacy, artificial intelligence, readability, health education

## Abstract

**Background:**

Online accessible patient education materials (PEMs) are essential for patient empowerment. However, studies have shown that these materials often exceed the recommended sixth-grade reading level, making them difficult for many patients to understand. Large language models (LLMs) have the potential to simplify PEMs into more readable educational content.

**Objective:**

We sought to evaluate whether 3 LLMs (ChatGPT [OpenAI], Gemini [Google], and Claude [Anthropic PBC]) can optimize the readability of PEMs to the recommended reading level without compromising accuracy.

**Methods:**

This cross-sectional study used 60 randomly selected PEMs available online from 3 websites. We prompted LLMs to simplify the reading level of online PEMs. The primary outcome was the readability of the original online PEMs compared with the LLM-simplified versions. Readability scores were calculated using 4 validated indices Flesch Reading Ease, Flesch-Kincaid Grade Level, Gunning Fog Index, and Simple Measure of Gobbledygook Index. Accuracy and understandability were also assessed as balancing measures, with understandability measured using the Patient Education Materials Assessment Tool-Understandability (PEMAT-U).

**Results:**

The original readability scores for the American Heart Association (AHA), American Cancer Society (ACS), and American Stroke Association (ASA) websites were above the recommended sixth-grade level, with mean grade level scores of 10.7,10.0, and 9.6, respectively. After optimization by the LLMs, readability scores significantly improved across all 3 websites when compared with the original text. Compared with the original website, Wilcoxon signed rank test showed ChatGPT improved the readability to 7.6 from 10.1 (*P*<.001); Gemini, to 6.6 (*P*<.001); and Claude, to 5.6 (*P*<.001). Word counts were significantly reduced by all LLMs, with a decrease from a mean range of 410.9-953.9 words to a mean range of 201.9-248.1 words. None of the ChatGPT LLM-simplified PEMs were inaccurate, while 3.3% of Gemini and Claude LLM-simplified PEMs were inaccurate. Baseline understandability scores, as measured by PEMAT-U, were preserved across all LLM-simplified versions.

**Conclusions:**

This cross-sectional study demonstrates that LLMs have the potential to significantly enhance the readability of online PEMs while maintaining accuracy and understandability, making them more accessible to a broader audience. However, variability in model performance and demonstrated inaccuracies underscore the need for human review of LLM output. Further study is needed to explore advanced LLM techniques and models trained for medical content.

## Introduction

Online health information is increasingly becoming a leading source for which US adults seek health advice. More individuals use online health information for health advice than family, friends, coworkers, health care professionals, or traditional media [1]. Health information sourced from online materials can supplement the patient-physician conversation regarding health advice [2], however, many individuals without access to a health care provider only seek information online [3]. This is concerning as online materials are not tailored to an individual patient’s specific needs and require readers to discern appropriately between valuable and less relevant health information [4]. Given that 36% of US adults have only basic or below basic health literacy skills [5], navigating the abundant health information online becomes particularly challenging.

Health literacy is an individual’s ability to understand and use health information to make informed decisions [6]. The inability to interpret health information can have serious consequences for patients, as individuals with low health literacy rates often experience poorer health outcomes [7]. They may also be more likely to be readmitted to the emergency department or access inappropriate health services than those with adequate health literacy, contributing to rising health care costs [8,9]. There is a growing recognition that the creators of health information must present materials in a simplified format that encourages understanding, as making patient education materials (PEMs) more readable and understandable has been shown to lead to better comprehension of health-related information among those with low health literacy [6,10]. Several validated measures of readability exist, including the Flesch Reading Ease [11] (FRE), Flesch-Kincaid Grade Level [12] (FKGL), Gunning Fog Index [13] (GFI), and the Simple Measure of Gobbledygook Index [14] (SMOGI). In addition, understandability can be measured by the Patient Education Materials Assessment Tool-Understandability [15] (PEMAT-U) scores.

The National Institutes of Health (NIH) recommends that PEM be written at a maximum reading level of sixth grade [16]. However, PEMs provided by hospitals and those available online are consistently above a sixth-grade reading level [17-19]. Further, one study reviewing 100 PEMs from 3 distinct PEM content vendors providing materials to the National Library of Medicine and electronic health records vendors found most of the PEMs were above an eighth-grade reading level [20]. A meta-analysis of 7891 websites using 13 different readability scales found mean grade level scores ranged from grade 10-15 [21]. Disease-specific studies focusing on stroke and heart failure found materials are consistently above the recommended reading level [22-24], which is especially concerning as they are among the leading causes of death in the United States [25]. While improvements in readability scores have been made over time [17] and human intervention can assist in improving readability [19], challenges remain in bringing readability to an appropriate level on a mass scale.

Large language models (LLMs) can consume massive amounts of data and perform a wide array of tasks that have made them useful tools to understand and generate text [26], making them an enticing option to improve the readability of PEMs. One study evaluated the impact of institutionally developed PEMs using LLMs and found that readability could be improved, but still did not meet the NIH recommended reading level [27]. In another study seeking to improve patient accessibility of published discharge summaries, LLMs enhanced readability and reliably reached the targeted reading level. However, the study concluded that LLM outputs required clinician oversight to ensure accuracy [28].

There are multiple publicly accessible LLMs available for use today, including ChatGPT (OpenAI) [29], Gemini (Google) [30], and Claude (Anthropic PBC) [31]. Potentially, patients or PEM creators could use these resources to simplify online PEM material, but little is known about the efficacy or safety of this approach, though risks of bias and transparency have been identified [32]. Existing literature on LLM simplification often focuses on institution-specific or topic-specific materials [33-35], which limits the broader applicability of the findings. These studies typically address readability improvements within a narrow context, leaving a gap in a holistic understanding of the impact on readability, understandability, and accuracy [27,33,36]. There is a need for research that evaluates PEMs across a wider range of health topics and sources to provide more generalizable insights. As such, the primary objective of this study was to evaluate if LLMs, when prompted, could simplify readability of online PEM content across a range of health topics and sources, while maintaining accuracy and understandability.

## Methods

### Overview

This cross-sectional study looked at online accessible PEMs accessed between July 1, 2024, and August 30, 2024. All study procedures complied with institutional ethical standards and those set by the Declaration of Helsinki and are reported using both the STROBE (Strengthening the Reporting of Observational Studies in Epidemiology; Multimedia Appendix 1) [37] reporting guidelines for cross-sectional studies and the TRIPOD-LLM (Transparent Reporting of a multivariable model for Individual Prognosis or Diagnosis–LLMs) checklist for reporting studies involving the use of LLMs (Multimedia Appendix 2) [38].

### Recruitment

The study used 60 randomly selected PEMs available online from 3 websites. The number 60 was chosen a priori based on feasibility. We selected 3 websites with publicly available health education materials: the American Heart Association [39] (AHA), the American Cancer Society [40] (ACS), and the American Stroke Association [41] (ASA). We selected these 3 organizations as heart disease, cancer, and stroke are among the leading causes of death in the United States [25]. Materials for review were selected from the organization’s website. A total of 20 articles from each site were randomly selected for review.

### Intervention

We chose to use free, publicly available generative artificial intelligence (AI) platforms to improve the readability of the 60 PEMs, and we accessed the platforms through the publicly available chat interfaces. We chose free platforms as they help to support equitable access to the technology for all patients; for this reason, we intentionally accessed the platforms through the same web-based interface that patients experience. These platforms were OpenAI’s ChatGPT, Google’s Gemini, and Anthropic’s Claude. Each of the 60 PEMs was entered into the freely available LLMs ChatGPT (Version: GPT-4, released on May 13, 2024, OpenAI), Gemini (Version: Gemini-1.5-flash, released on May 24, 2024, Google), and Claude (Version: Claude 3.5 Sonnet, released on June 20, 2024, Anthropic).

We asked each LLM via prompt to “Translate to a fifth-grade reading level” and pasted the original article text into the LLM. We selected this prompt to model research showing that AI tools improved readability by targeting a lower level than the sixth-grade level recommended by the NIH. This approach, which has been proven successful, was designed to account for variability in grade-level interpretations by LLMs [27]. We then saved copies of each output from the LLM, resulting in 4 total versions of each material (the original plus 3 LLM versions).

### Measures

The primary outcome of readability of each PEM and the LLM-simplified PEM was measured by online accessible scoring of FRE, FKGL, GFI, and SMOGI [42]. The PEMAT-U score was manually scored by a project team member using the PEMAT guide [15]. We also extracted the number of words for each PEM and each LLM-simplified PEM. We completed assessments for errors or inaccuracies by reviewing the LLM-simplified PEM and comparing it to the original PEM as the criterion standard. A total of 2 project team members in nonclinical roles completed these reviews independently. If either team member marked the LLM-simplified PEM as having errors or inaccuracies, a physician member of the project team reviewed the article to determine the final outcome. Errors or inaccuracies were defined as instances in which the original message was changed, or the LLM simplified content misrepresented the meaning of the original content which could lead to a different interpretation by the reader.

### Statistical Analysis

Descriptive statistics presented include means and SD of the FRE, FKGL, GFI, SMOGI, and PEMAT-U scores, as well as the number of words to measure material length independently of the score. We used a 1-way ANOVA test to test for significant differences in the original PEM of the material between websites. To assess statistically significant differences in readability scores and word count from the original PEM to the LLM-simplified PEM, we used Wilcoxon signed rank test to compare each LLM-simplified PEM to its corresponding original PEM. All data were prepared and analyzed in IBM SPSS (version 28.0.1.1) [43].

### Ethical Considerations

The project does not involve human participants in the research and does not require institutional review.

## Results

We evaluated 60 unique PEMs from 3 different websites (Table S1 in Multimedia Appendix 3). Mean readability scores across the 3 websites were not significantly different from each other, regardless of the readability used. The AHA had the highest FKGL, GFI, and SMOGI reading scores, and the lowest FRE score, though none were significantly different. The ACS had significantly more words than both the AHA and ASA.

All 3 LLMs had significantly improved readability scores when compared with the original source. Gemini and Claude had greater improvements in readability than ChatGPT for each website. Though improved, there were only 2 instances in which the project target of achieving a mean sixth-grade reading level was met (Chat GPT’s AHA FKGL score and Gemini’s ACA FKGL and GFI score and ASA GFI score). There were 5 instances in which the LLM prompt of achieving a mean fifth-grade reading level was met (ChatGPT’s ASA FKGL score, Gemini’s AHA GFI score, Claude’s AHA GFI score, and Claude’s ACS and ASA GFI score). In 5 other instances, the mean reading level was improved beyond a fifth-grade reading level (Gemini’s AHA and ASA FKGL score, and Claude’s AHA, ACS, and ASA FKGL score). Readability scores and SD for each website’s original PEM and the 3 LLM-simplified PEM are shown in Table 1.

**Table 1:** Mean reading scores and SD of the original version of the patient education material and each large language model version (N=60).

Website and score			Original		ChatGPT					Gemini					Claude		
					Value, mean (SD)		*P* value^a^		Value, mean (SD)			*P* value		Value, mean (SD)			*P* value
**American Heart Association**																	
	FRE^b^	52.9 (11)		74.2 (7.4)		<.001		86.3 (7.8)			<.001		83.6 (6.3)			<.001	
	FKGL^c^	9.1 (1.7)		6.1 (1.3)		<.001		3.9 (1.3)			<.001		3.8 (0.9)			<.001	
	GFI^d^	11.3 (2)		8.0 (1.3)		<.001		5.8 (1.1)			<.001		5.6 (1)			<.001	
	SMOGI^e^	11.7 (1.4)		9.1 (1.1)		<.001		7.3 (1)			<.001		7.3 (0.8)			<.001	
	PEMAT-U^f^	75.5 (13.1)		74.7 (11.4)		.78		81.1 (8.5)			.15		85.4 (5.8)			.01	
	Number of words	663.0^f^ (286.6)		213.3 (54.1)		<.001		213.3 (54.1)			<.001		243.5 (65.6)			<.001	
**American Cancer Society**																	
	FRE	59.7 (9)		65.8 (10.3)		<.001		67.1 (12.3)			.009		79.4 (9.2)			<.001	
	FKGL	8.5 (1.5)		7.1 (1.5)		<.001		6.6 (1.8)			<.001		4.5 (1.3)			<.001	
	GFI	10.2 (1.6)		8.1 (1.5)		<.001		7.7 (2)			<.001		5.6 (1)			<.001	
	SMOGI	11.1 (1.3)		9.8 (1.3)		<.001		9.4 (1.4)			<.001		7.5 (1.1)			<.001	
	PEMAT-U	81.3 (11.1)		76.5 (10.1)		.04		87.6 (5.8)			.03		81.6 (6.8)			.88	
	Number of words	953.9^g^ (725.5)		243.6 (102.4)		<.001		243.6 (102.4)			<.001		248.1 (60.5)			<.001	
**American Stroke Association**																	
	FRE	59 (14.6)		75 (9.2)		<.001		80.5 (9.5)			<.001		85.6 (6)			<.001	
	FKGL	7.9 (2.3)		5.6 (1.5)		<.001		4.2 (1.4)			<.001		3.6 (1.3)			<.001	
	GFI	10 (2.6)		7.4 (1.7)		<.001		6.0 (1.3)			<.001		5.6 (1.5)			<.001	
	SMOGI	10.8 (2)		8.7 (1.7)		<.001		7.8 (1.2)			<.001		7.1 (1.4)			<.001	
	PEMAT-U	77.5 (9.3)		75.9 (8.3)		.26		82.8 (4.9)			.05		78.9 (6.6)			.72	
	Number of words	410.9^g^ (207.4)		201.9 (75.9)		<.001		201.9 (75.9)			<.001		230.8 (66.1)			<.001	

^a^*P* values reported are the large language model text compared with original source text, using Wilcoxon signed rank test.

^b^FRE: Flesch Reading Ease.

^c^FKGL: Flesch-Kincaid Grade Level.

^d^GFI: Gunning-Fog Index.

^e^SMOGI: Simple Measure of Gobbledygook Index,

^f^PEMAT-U: Patient Education Materials Assessment Tools–Understandability.

^g^Results of 1-Way ANOVA test indicate original sites are significantly different from each other at *P*<.05.

**Figure 1:**
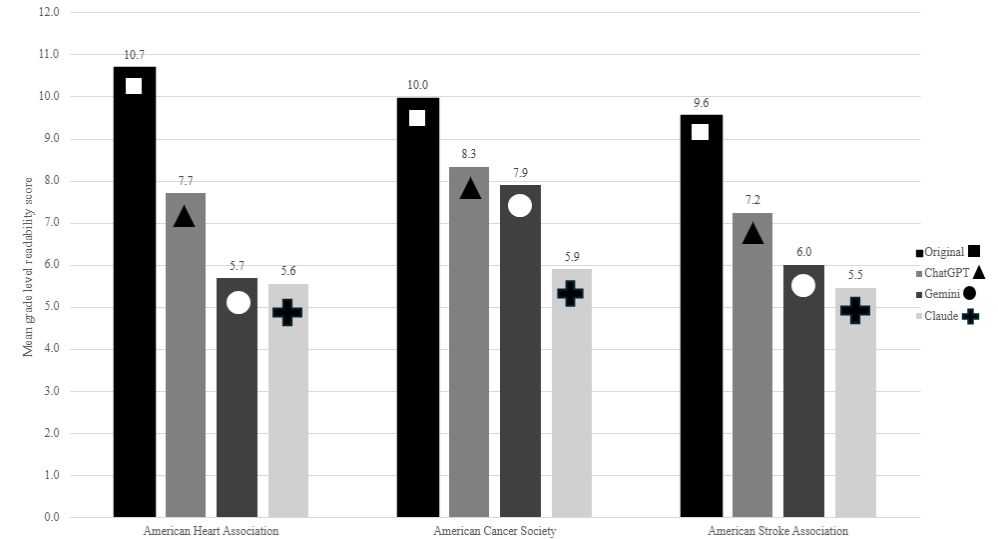
Mean grade level scores for the original version of the patient education material and each large language model version (N=60).

We calculated the average of the 3 grade-level readability scores (FKGL, GFI, and SMOGI) for each PEM version of the material, and compared them across sites. Compared with the original website, ChatGPT improved the readability to 7.6 from 10.1 (*P*<.001); Gemini, to 6.6 (*P*<.001); and Claude, to 5.6 (*P*<.001). Of LLM-simplified versions of the PEM, only Claude consistently achieved a mean fifth-grade reading level on all 3 websites (5.5-5.9), while Gemini did on one of the websites, and ChatGPT on none. Figure 1 shows mean readability scores by website and PEM version.

We assessed accuracy for all LLM-simplified PEM content based on its original version. ChatGPT had no inaccuracies on all 3 sites, while Gemini experienced 2 inaccuracies on the AHA website and Claude experienced one inaccuracy on the ACA and one on the ASA website. Accuracy data is presented in Table 2. The 4 inaccuracies identified are presented in Table 3, with the original and LLM-simplified text.

**Table 2:** Number of inaccuracies and inaccuracy rate of each large language model version when compared with the original, by large language model.

Website	ChatGPT, n (%)	Gemini, n (%)	Claude, n (%)
American Heart Association (N=20)	0 (0)	2 (10)	0 (0)
American Cancer Society (N=20)	0 (0)	0 (0)	1 (5)
American Stroke Association (N=20)	0 (0)	0 (0)	1 (5)
Total (N=60)	0 (0)	2 (3.3)	2 (3.3)

**Table 3:** Examples of inaccuracies in the large language model version when compared with the original content. The text in italics highlights the key difference and issue with the text.

Patient education material site	Original text	Large language model text	Large language model
American Heart Association	Coronary microvascular disease Mitral valve stenosis	Tiny heart trouble A sticky valve	Gemini
American Cancer Society	Solitary papillomas (solitary intraductal papillomas) are single tumors that often grow in the large milk ducts near the nipple. They are a common cause of clear or bloody nipple discharge, especially when it comes from only one breast. They may be felt as a small lump behind or next to the nipple. *Sometimes they can be painful.*	Sometimes, small bumps can grow inside the tiny pipes in your breast that carry milk. These bumps are not dangerous *and won't hurt you.* They’re called intraductal papillomas.	Claude
American Stroke Association	Bathroom transfers: Practice *transfers with your therapist* first so you’re confident and safe.	Moving safely: Practice moving with *your helper* first.	Claude

## Discussion

### Principal Findings

Our study reveals that the readability of PEMs from the AHA, ACS, and ASA websites significantly exceeds the NIH-recommended sixth-grade reading level. This finding aligns with existing literature, which consistently reports that online PEMs are often written at a higher reading level [21-24]. The mean readability scores for the original PEMs were 10.7, 10, and 9.6 for AHA, ACS, and ASA, respectively.

The application of LLMs such as ChatGPT, Gemini, and Claude demonstrated a substantial improvement in the readability of these materials. Still, despite being prompted to translate the text to a fifth-grade reading level, the LLMs often produced content that was above this target. ChatGPT had the highest mean reading level (7.2-8.3), followed by Gemini (5.7-7.9). Only Claude consistently achieved a mean fifth-grade reading level (5.5-5.9), which is below the NIH recommended maximum level. These results suggest that while LLMs can enhance readability, achieving the desired reading level remains challenging. Alternative prompting techniques that include more description or seek a lower reading level score may yield different results. Although no specific patterns related to content or type of PEM were identified that scored higher or lower, FKGL readability scores were consistently lower than GFI or SMOGI scores, highlighting the variation of the readability scoring algorithms.

Understandability, as measured by the PEMAT-U, saw some significant improvements, though this was not consistent across all sites and LLMs. Some notable improvements within the PEMAT-U by the LLMs focused on word choice by only using medical terms to familiarize the audience and subsequently defining them, organization by breaking the material into short sections if the original material was not, and layout by adding bullet points.

Interestingly, our study found that Google’s Gemini outperformed ChatGPT, contrary to previous research where ChatGPT was more effective than Google’s Bard [27]. This variability is not unexpected, as newer models can exhibit different performance levels, but it does highlight the need for further research to identify the most reliable and effective LLM-based approaches for optimizing PEM readability.

Accuracy is another critical factor when simplifying PEMs. Our study found high accuracy rates for LLM-simplified PEMs, with ChatGPT achieving 100% accuracy and Gemini and Claude at 96.7%. These rates are significantly higher than those reported in previous work on simplified discharge summaries, which had a 54% accuracy rate [28]. The higher accuracy in this study may be attributed to the simpler nature of PEMs compared with the more technically worded discharge summaries. However, the tradeoff between simplification and accuracy remains a concern in this study as well, as Gemini and Claude, the models that performed best at simplification, were the least accurate.

Beyond accuracy concerns, the use of AI in health care presents some inherent risks. One meta-analysis found bias and transparency are 2 primary risks associated with AI in health care [32]. This is an important consideration when using LLMs to simplify PEMs, as important content intended to minimize bias in PEM may be omitted for the sake of simplification. In addition, LLMs are not obviously transparent about the source of data used in their text generation, presenting risks of misinformation and the need for clinical oversight.

Overall, this study demonstrates that while publicly available LLMs hold great promise, they may not yet be fully reliable for direct use by patients to simplify PEMs. However, based on our results, PEM creators who are content experts in their respective fields could certainly start to use LLMs to improve the readability of their own materials. This could be accomplished before the publication of materials online through prompt engineering and human review of the simplified PEM. To gain efficiency, content creators could prompt LLMs to create PEM rather than simplify it and add guardrails to ensure necessary content is maintained. This approach could help address the persistent issue of PEMs being written above the recommended reading level, which disproportionately affects individuals with low health literacy. Given the increasing reliance on online information as a primary source of health information, the adoption of LLMs by online PEM creators has great potential for enhancing patient understanding and improving health outcomes.

### Recommendations

Future research should explore more advanced LLM engineering techniques, such as few-shot learning, where LLMs learn through examples included within the prompt to achieve targeted readability goals [28]. One could envision a library of prompts made available through a publicly accessible application designed specifically for this purpose. In addition, integrating LLM self-evaluation, where the model checks its own output for inaccuracies, and generating knowledge prompting, which involves asking the model to generate its knowledge about a topic before providing an answer to reduce hallucinations and improve accuracy, could further enhance the effectiveness of these models. While our study focused on free, publicly accessible LLMs to ensure equitable access, more sophisticated pay-for-play models like OpenAI’s o1 model and Claude Pro may offer greater reliability and effectiveness. In addition, LLMs trained specifically on medical data or fine-tuned for this specific task are promising avenues for future study.

While the out-of-the-box use of LLMs is not yet fully reliable for direct patient use, the significant opportunity for improved performance suggests that this capability is well within reach. With continued advancements and refinements, we are confident that LLM simplification of PEMs can become a safe and effective patient-facing tool that empowers patients to better understand their health conditions and ultimately improves health literacy and outcomes.

### Limitations

This study has several limitations. First, it only assessed content from 3 websites, and results may vary with other sources of PEM, especially PEM on rare diseases, where online PEM may be less abundant. Second, the study included only English-language PEMs; materials in other languages may yield different outcomes. Third, we used publicly available content and prompted the LLMs to translate the content to a specific reading level. Different methods, such as asking LLMs to create content from scratch, may produce different results. In addition, the manual calculation of PEMAT-U scores may introduce subjective bias, and the focus on readability and understandability of text ignores the capability of LLMs to create diagrams, infographics, and figures that can help with patient understanding. Further, team members calculating the PEMAT-U scores and accuracy were not blinded to the LLM they were evaluating due to limited project resources, introducing reviewer bias. Finally, while the content was generally accurate, we did not evaluate whether important omissions were made during simplification. Future studies should assess the impact of content loss on the PEMs’ educational value. Although independent reviews of the materials were completed by nonclinical team members to prevent medical knowledge bias in the evaluation of PEMAT-U and accuracy, it’s possible reviews by patients or patient caregivers would respond differently.

### Conclusions

Online PEMs consistently exceed the NIH-recommended maximum sixth-grade reading level, posing a challenge for individuals with low health literacy. LLMs offer a promising solution for simplifying PEMs to improve accessibility. Our study demonstrates that LLMs can significantly enhance readability while maintaining high accuracy. However, achieving the desired reading level remains challenging, and human oversight is necessary to ensure the completeness and accuracy of simplified content. Further research is needed to explore advanced LLM techniques and models specifically trained for medical content to optimize the readability and understandability of PEMs.

## Appendix

Multimedia Appendix 1 STROBE (Strengthening the Reporting of Observational Studies in Epidemiology) reporting guidelines checklist for cross-sectional studies.

Multimedia Appendix 2 TRIPOD-LLM (Transparent Reporting of a multivariable model for Individual Prognosis or Diagnosis-Large Language Models) checklist.

Multimedia Appendix 3 Websites and articles titles of online patient education materials utilized in this study, including the number of words, readability, and understandability scores for each article (N = 60).

## Abbreviations

ACS: American Cancer Society
AHA: American Heart Association
AI: artificial intelligence
ASA: American Stroke Association
FKGL: Flesch-Kincaid Grade Level
FRE: Flesch Reading Ease
GFI: Gunning Fog Index
LLM: large language model
NIH: National Institutes of Health
PEM: patient education material
PEMAT-U: Patient Education Materials Assessment Tool-Understandability
SMOGI: Simple Measure of Gobbledygook Index
STROBE: Strengthening the Reporting of Observational Studies in Epidemiology
TRIPOD-LLM: Transparent Reporting of a multivariable model for Individual Prognosis or Diagnosis-Large Language Models

## Data Availability

The datasets generated or analyzed during this study are available from the corresponding author on reasonable request.
