# Supplementary material for: Enhancing the Readability of Online Patient Education Materials Using Large Language Models: Cross-Sectional Study
Source: J Med Internet Res. 2025 Jun 4;27:e69955. doi: 10.2196/69955 (PMC12177420; doi:10.2196/69955)
Supplement: Multimedia Appendix 3 [file jmir_v27i1e69955_app3.docx]

**Table S1.** Websites and articles titles of online patient education materials utilized in this study, including the number of words, readability, and understandability scores for each article (N = 60).

| Website | Article Number | Article Title | Number of Words | FRE | FKGL | GFI | SMOGI | PEMAT-U |
| --- | --- | --- | --- | --- | --- | --- | --- | --- |
| American Heart Association | 1 | Protect Your Heart in the Heat | 727 | 68.7 | 6.5 | 6.5 | 9.8 | 87.5% |
|  | 2 | Living with Diabetes | 932 | 60.7 | 7.6 | 7.6 | 10.7 | 81.3% |
|  | 3 | Smoking and Heart Disease in Women | 539 | 67.9 | 7.4 | 7.4 | 10.5 | 93.8% |
|  | 4 | Coronary Heart Disease - Coronary Heart Disease | 337 | 49.1 | 9.6 | 9.6 | 12.5 | 75.0% |
|  | 5 | Roles of Your Four Heart Valves | 391 | 55.8 | 8.0 | 8.0 | 11.4 | 93.8% |
|  | 6 | What is Diabetes? | 848 | 44 | 10.3 | 10.3 | 13.0 | 81.3% |
|  | 7 | Risks for Heart Valve Problems | 351 | 51.5 | 10.2 | 10.2 | 12.5 | 66.7% |
|  | 8 | Recurrent Pericarditis | 990 | 42.7 | 10.3 | 10.3 | 12.0 | 75.0% |
|  | 9 | Sleep Disorders and Atrial Fibrillation | 503 | 41.9 | 11.9 | 11.9 | 14.2 | 87.5% |
|  | 10 | Coronary Microvascular Disease (MVD) | 1249 | 45.6 | 9.7 | 9.7 | 11.5 | 62.5% |
|  | 11 | Ross Procedure | 226 | 57 | 8.3 | 8.3 | 11.2 | 80.0% |
|  | 12 | Problem: Mitral Valve Stenosis | 443 | 48 | 10.0 | 10.0 | 12.7 | 66.7% |
|  | 13 | The flue vaccine: What you need to know this year | 314 | 60.5 | 8.7 | 8.7 | 10.9 | 84.6% |
|  | 14 | Understanding Your Risks for Excessive Blood Clotting | 647 | 55.4 | 8.5 | 8.5 | 11.8 | 76.9% |
|  | 15 | Preventing and Managing Falls in Adults with Cardiovascular Disease | 662 | 36.2 | 11.6 | 11.6 | 13.2 | 56.3% |
|  | 16 | What is Cardiovascular Disease? | 898 | 62.2 | 6.9 | 6.9 | 9.9 | 66.7% |
|  | 17 | Illegal Drugs and Heart Disease | 589 | 41.3 | 10.6 | 10.6 | 12.7 | 69.2% |
|  | 18 | Syncope (Fainting) | 1121 | 34.4 | 12.1 | 12.1 | 14.4 | 43.8% |
|  | 19 | Peripheral Angiography | 879 | 68.9 | 6.7 | 6.7 | 10.1 | 69.2% |
|  | 20 | Causes and Prevention of Heart Disease for Women | 614 | 66.8 | 7.1 | 7.1 | 9.8 | 92.3% |
| American Cancer Society | 21 | Risk Factors and Causes of Childhood Cancer | 595 | 62.8 | 8.2 | 8.2 | 10.5 | 85.0% |
|  | 22 | Limitations of Mammograms | 753 | 55 | 9.3 | 9.3 | 12.1 | 83.0% |
|  | 23 | Intraductal Papillomas of the Breast | 411 | 53.2 | 9.5 | 9.5 | 12.1 | 73.0% |
|  | 24 | Anal Cancer Survivor Rates | 536 | 58.4 | 8.5 | 8.5 | 11.2 | 92.0% |
|  | 25 | Can Rhabdomyosarcoma Be Found Early? | 296 | 56.7 | 9.4 | 9.4 | 11.5 | 91.0% |
|  | 26 | Radiation Therapy for Colorectal Cancer | 1172 | 48.7 | 10.2 | 10.2 | 12.1 | 58.0% |
|  | 27 | Signs and Symptoms for Rhabdomyosarcoma | 372 | 68.2 | 7.9 | 7.9 | 10.5 | 89.0% |
|  | 28 | Surgery to Treat Lung Carcinoid Tumors | 832 | 57.5 | 9.3 | 9.3 | 12.5 | 83.0% |
|  | 29 | Cancer of Unknown Primary Stages | 590 | 67 | 7.8 | 7.8 | 10.4 | 100.0% |
|  | 30 | Targeted Therapy Drugs for Acute Myeloid Leukemia (AML) - ACS Appension | 1657 | 57.9 | 9.3 | 9.3 | 11.6 | 66.7% |
|  | 31 | Questions to Ask Your Doctor About Small Intestine Cancer | 585 | 78.6 | 4.7 | 4.7 | 8.0 | 91.0% |
|  | 32 | Hormone Therapy for a Cancer of Unknown Primary | 353 | 41.3 | 11.0 | 11.0 | 13.7 | 73.0% |
|  | 33 | Treatment for Uterine Sarcoma, by Type and Stage | 1314 | 50.3 | 10.0 | 10.0 | 12.4 | 67.0% |
|  | 34 | Protecting People in Clinical Trials | 795 | 58.3 | 8.5 | 8.5 | 11.5 | 82.0% |
|  | 35 | Precision or Personalized Medicine | 2243 | 62.5 | 8.1 | 8.1 | 10.7 | 67.0% |
|  | 36 | Social Security Disability Insurance for People with Cancer | 846 | 54.1 | 8.5 | 8.5 | 10.9 | 91.0% |
|  | 37 | If You Have Laryngeal or Hypopharyngeal Cancer | 3133 | 74.6 | 6.0 | 6.0 | 8.6 | 87.0% |
|  | 38 | HPV Signs and Symptoms | 576 | 70.1 | 7.2 | 7.2 | 10.4 | 73.0% |
|  | 39 | Cancer Facts for Men | 1592 | 65.2 | 7.2 | 7.2 | 10.0 | 83.0% |
|  | 40 | American Cancer Society Recommendations for Prostate Cancer Early Detection | 427 | 54.2 | 10.1 | 10.1 | 12.2 | 91.0% |
| American Stroke Association | 41 | Hemiparesis | 635 | 48.6 | 8.6 | 8.6 | 10.6 | 82.0% |
|  | 42 | Cryptogenic Stroke or Stroke of Unknown Cause | 354 | 47.7 | 10.0 | 10.0 | 12.6 | 92.0% |
|  | 43 | 15 Things Caregivers Should Know After a Loved One Has Had a Stroke | 665 | 49.3 | 9.4 | 9.4 | 13.1 | 84.6% |
|  | 44 | Trouble Swallowing After Stroke (Dysphagia) | 480 | 63.8 | 6.8 | 6.8 | 9.9 | 83.3% |
|  | 45 | Choosing the Right Stroke Rehab Facility | 929 | 39.3 | 10.7 | 10.7 | 12.6 | 87.5% |
|  | 46 | Effects of Stroke | 445 | 66.4 | 6.6 | 6.6 | 9.5 | 80.0% |
|  | 47 | Auditory Overload | 157 | 53.5 | 7.9 | 7.9 | 10.5 | 83.3% |
|  | 48 | Brain Stem Stroke | 296 | 57.8 | 8.9 | 8.9 | 11.8 | 60.0% |
|  | 49 | Talking to Your Doctor | 607 | 66.6 | 6.8 | 6.8 | 10.3 | 80.0% |
|  | 50 | Hearing Disturbances After Stroke | 205 | 53.3 | 7.9 | 7.9 | 10.4 | 83.3% |
|  | 51 | Spatial Neglect | 323 | 66.3 | 7.5 | 7.5 | 10.1 | 58.3% |
|  | 52 | Bathing Tips for Stroke Survivors | 533 | 82.1 | 4.3 | 4.3 | 7.4 | 86.7% |
|  | 53 | Depression and Stroke | 337 | 41.2 | 9.9 | 9.9 | 12.4 | 66.7% |
|  | 54 | Aphasia Therapy When Insurance Stops | 282 | 53.1 | 9.0 | 9.0 | 12.2 | 66.7% |
|  | 55 | Claw Toe | 511 | 60.9 | 7.9 | 7.9 | 11.0 | 75.0% |
|  | 56 | Aspirin and Stroke | 529 | 54 | 8.9 | 8.9 | 11.9 | 80.0% |
|  | 57 | Palliative Care | 283 | 36.6 | 12.1 | 12.1 | 14.6 | 66.7% |
|  | 58 | Shopping Tips for Stroke Survivors | 125 | 77.1 | 5.1 | 5.1 | 8.8 | 80.0% |
|  | 59 | One-Sided Neglect | 425 | 69.1 | 6.9 | 6.9 | 10.5 | 75.0% |
|  | 60 | Goal Setting Worksheet | 96 | 94.1 | 1.8 | 1.8 | 6.2 | 80.0% |

FRE Flesch Reading Ease, FKGL Flesch-Kincaid Grade Level, GFI Gunning-Fog Index, SMOGI Simple Measure of Gobbledygook Index, PEMAT-U Patient Education Materials Assessment Tools - Understandability
